# Identification and validation of CKAP2 as a novel biomarker in the development and progression of rheumatoid arthritis

**DOI:** 10.3389/fimmu.2025.1606201

**Published:** 2025-06-25

**Authors:** Qiongbing Zheng, Youmian Lan, Jiexin Chen, Ling Lin

**Affiliations:** ^1^ Department of Rheumatology and Immunology, First Affiliated Hospital of Shantou University Medical College, Shantou, China; ^2^ Department of Neurology, Shantou Central Hospital, Shantou, China; ^3^ Department of Endocrinology and Metabolism, First Affiliated Hospital of Shantou University Medical College, Shantou, China; ^4^ Department of Rheumatology, Shantou University Medical College, Shantou, China

**Keywords:** rheumatoid arthritis, machine learning, bioinformatics analysis, mendelian randomization, CKAP2, fibroblast-like synoviocytes

## Abstract

**Background:**

Rheumatoid arthritis (RA) is a common chronic joint disease. However, many patients exhibit inadequate responses to treatment due to disease heterogeneity. Identifying novel biomarkers for RA is crucial for advancing molecular diagnosis and identifying potential therapeutic targets.

**Methods:**

Synovial tissue transcriptome data from RA patients and healthy controls were obtained from the GEO database. Differentially expressed gene (DEG) analysis, functional enrichment analysis, and weighted gene co-expression network analysis (WGCNA) identified key gene modules in RA. Machine learning algorithms were used to identify hub genes, followed by immune infiltration analysis and gene set variation analysis (GSVA). Mendelian randomization (MR) analysis explored the causal relationship between hub genes and RA. Clinical synovial tissue samples were used to validate CKAP2 expression via quantitative real-time polymerase chain reaction (qRT-PCR), western blot, and immunohistochemistry (IHC). The potential role of CKAP2 in the pathogenesis of RA was investigated through CCK-8 assay, wound healing assay, transwell migration assay and transwell invasion assay.

**Results:**

A total of 242 DEGs were identified between 20 RA patients and 17 healthy controls. Six gene modules were recognized, with the “turquoise” module associated with RA (cor = 0.39, *p* = 0.00017). Three hub genes, *CKAP2* (AUC = 0.876), *POU2AF1* (AUC = 0.885), and *HLA-DOB* (AUC = 0.897), involved in the IL-6/JAK/STAT3 signaling pathway and inflammation, were identified. Immune infiltration analysis showed these genes were associated with plasma cells and T cell infiltration. MR analysis confirmed a causal relationship between *CKAP2* and RA. qRT-PCR, western blot, and IHC demonstrated CKAP2 expression was higher in RA synovial tissue compared to osteoarthritis (OA) samples. Cellular functional assays demonstrated that CKAP2 knockdown inhibited the proliferation, migration, and invasion of MH7A and HFLS-RA cells.

**Conclusion:**

*CKAP2*, a signature gene in RA, is highly expressed in RA synovial tissues, and contributes to the pathogenesis of RA by promoting the proliferation, migration, and invasion of MH7A and HFLS-RA cells. This gene holds potential as a novel biomarker for RA and provides valuable insights into its molecular diagnosis and targeted therapies.

## Introduction

Rheumatoid arthritis (RA) is a systemic inflammatory autoimmune disease that affects about 0.5%~1% of the population, with women being 3~5 times more likely to be affected than men (1–3). The pathogenesis of RA is multifactorial, involving genetic, environmental, and immunologic factors that contribute to both initiation and progression of the disease (4, 5). These processes lead to synovial inflammation and hyperplasia, which ultimately results in joint destruction, swelling, pain, and symmetric polyarthritis, impairing the patient’s quality of life (6–8). Although current anti-cytokine therapies have improved disease control and prognosis, the complexity of pathogenesis and its heterogeneity still results in many patients experiencing inadequate responses to treatment (9, 10). Thus, identifying novel RA biomarkers and developing more effective molecular diagnostic and therapeutic strategies remain of paramount importance.

The advent of gene microarray technology and high-throughput techniques has made bioinformatics methods crucial for the effective identification of differentially expressed genes (DEGs) (11). In recent years, machine learning has been increasingly utilized to address complex challenges in the biomedical field. The integration of bioinformatics analysis with machine learning presents valuable opportunities to enhance the accuracy, reliability, and predictability of disease diagnosis (12–14). Mendelian randomization (MR) is a method that overcomes the limitations of observational studies to identify potential causal relationships between exposure and outcome, making it a widely used tool in causal association research (15, 16).

Cytoskeleton-associated protein 2 (CKAP2) encodes a protein that stabilizes microtubules and plays a crucial role in the regulation of cytokinesis, which is closely linked to cellular proliferative activity, migration, and invasion (17–19). Previous studies have shown that CKAP2 expression is elevated in various cancers, including gastric adenocarcinoma, lung adenocarcinoma, hepatocellular carcinoma, cervical carcinoma, and breast carcinoma, where it influences tumor cell proliferation, migration and invasion (20–24). In the progression of RA, proliferation, migration and invasion of fibroblast-like synoviocytes (FLS) are key factors contributing to synovial hyperplasia and joint destruction (25, 26). However, the role of CKAP2 in RA is unknown and whether CKAP2 can participate in proliferation, migration and invasion in FLS yet to be investigated.

In this study, we employed machine learning algorithms combined with bioinformatics analysis and MR analysis to identify genes causally associated with RA. The expression of these hub genes in RA was subsequently validated using clinical tissue samples, and their potential role in RA pathogenesis was further explored through cellular functional assays. This research aimed to identify novel biomarkers for RA and provide new insights into its molecular diagnosis and targeted therapies.

## Materials and methods

### Data collection and processing

The microarray dataset for RA was retrieved from the GEO database of the National Center for Biotechnology Information (NCBI) (http://www.ncbi.nlm.nih.gov/geo/) using the search term “rheumatoid arthritis.” The selection criteria were as follows: (1) the study type involved expression profiling via microarrays, (2) the tissue type was synovial tissue, and (3) the organism was *Homo sapiens*. To minimize the influence of biological disease-modifying anti-rheumatic drugs (bDMARDs), only pre-treatment samples were included (27, 28). In total, four gene expression datasets (GSE206848, GSE55235, GSE172188, and GSE12021) were identified as suitable. The GSE206848, GSE55235, and GSE172188 datasets were used as the training set, consisting of 20 RA patients and 17 healthy controls, while the GSE12021 dataset served as the validation set, comprising 12 RA patients, 9 healthy controls, and 10 osteoarthritis (OA) patients. Information on patient age and sex is provided in **Supplementary Table S1**.

The “GEOquery” package was employed to convert the probe matrix into a gene expression matrix, using probe annotation files. If multiple probes corresponded to the same gene, the expression values of the first probe were retained. Given that these three datasets were derived from different platforms and exhibited batch effects, the “sva” package was utilized to correct for platform-related batch effects.

### Identification of DEGs and functional enrichment analysis

The “limma” package was employed to analyze DEGs between RA patients and healthy controls. The selection criteria for DEGs included a *p* < 0.05 and an absolute fold change > 2. Functional enrichment analysis of Gene Ontology (GO) terms and Kyoto Encyclopedia of Genes and Genomes (KEGG) pathways for the upregulated DEGs was performed using the “clusterProfiler” package in R. Pathways with *p* < 0.05 was considered significantly enriched.

### Weighted gene co-expression network analysis for the DEGs

We used the R package “WGCNA” to construct a weighted gene co-expression network for the DEGs. The soft threshold was selected using the “pickSoftThreshold” algorithm to build the gene co-expression modules. Each module contained at least 5 genes, with any remaining ungrouped genes assigned to the grey module. We calculated the correlation coefficients between modules and phenotypes to identify modules closely associated with RA. Additionally, gene significance and module membership were assessed to evaluate the relationship between gene modules and RA patients.

### Screening of hub genes by machine learning algorithms

We employed machine learning algorithms for hub gene selection, including the random forest (RF) model (29) and least absolute shrinkage and selection operator (LASSO) regression (14).

The RF model is a decision tree-based machine learning approach. In this study, we utilized R package “randomForest” to build the random forest model. To determine the optimal number of variables, we computed the average error rate of candidate module genes. We then evaluated the error rate across a range of tree numbers, from 1 to 1000, and selected the number of trees that yielded the lowest error rate. Finally, we determined the feature importance scores for each candidate gene in the significant modules and selected genes with importance values greater than 0.

LASSO regression is a widely used machine learning algorithm for fitting generalized linear models. By applying an L1 penalty (λ), coefficients of less important variables are set to zero, thereby selecting the most important variables and building optimal classification models. In our study, we performed LASSO regression analysis on the candidate genes using R package “glmnet” to identify hub genes. The optimal value of λ was determined through tenfold cross-validation, selecting the value that resulted in the smallest standard error.

### Diagnostic value of hub genes in RA

To evaluate the diagnostic accuracy of hub genes selected through machine learning algorithms, we employed the R package “pROC” to plot receiver operating characteristic (ROC) curves comparing RA patients with healthy controls in the training set. The area under the curve (AUC) was used to assess the diagnostic accuracy of these genes as potential hub genes for RA, with a larger AUC indicating higher accuracy. This analysis was subsequently validated in the independent validation set. Furthermore, to assess the specificity of the hub genes for RA diagnosis, we plotted ROC curves comparing RA patients to OA controls in the validation set. Expression levels of the hub genes in RA patients and controls were visualized using box plots generated by R package “ggplot2.”

### Gene set variation analysis and immune infiltration analysis of hub genes

We conducted gene set variation analysis using the R package “GSVA” to explore the correlation between hub genes and hallmark signaling pathways. Subsequently, we applied the CIBERSORT algorithm to determine the infiltration of different immune cells in RA synovial tissues and healthy controls. Spearman correlation coefficients were then used to analyze the relationships between hub genes and immune cell types.

### Identification of the causal relationship between signature genes and RA via MR analysis

#### Data source

In this study, we performed MR analysis using data from the IEU Open GWAS database (https://gwas.mrcieu.ac.uk). It is important to note that the participants in the studies included in the IEU Open GWAS database provided informed consent. GWAS data for the *CKAP2*, *POU2AF1*, and *HLA-DOB* genes were obtained from the following GWAS IDs: eqtl-a-ENSG00000136108, eqtl-a-ENSG00000110777, and eqtl-a-ENSG00000241106, respectively. Additionally, the UC GWAS database (GWAS ID: ebi-a-GCST90018910) contributed data for 417,256 individuals of European ancestry, including 8,255 RA patients and 409,001 healthy controls.

#### Instrumental variables selection

In MR analysis, IVs derived from genetic variants are used to obtain unbiased estimates of the causal effects of the exposure variable on the outcome variable. To begin, we identified single nucleotide polymorphisms (SNPs) that were significantly associated with the exposure variable (*p* < 5×10^-8^) as IVs for MR analysis. SNPs in linkage disequilibrium (LD) within a 10,000 kb distance and with an R² < 0.01 were excluded. The strength of the IVs was assessed using the F-statistic: F = (N- k-1)R²/[k(1 - R²)], where R² represents the proportion of variance in the exposure explained by each IV. An F-value greater than 10 indicates sufficient statistical power to retain the instrument.

#### MR analysis

To validate the causal relationship between exposure and outcome, our study employed R package “TwoSampleMR” for two-sample MR analysis, utilizing several MR methods, including inverse variance-weighted (IVW), weighted median (WM), MR-Egger, simple mode, and weighted mode. The IVW method was primarily used due to its superior statistical efficiency compared to the other methods and its ability to consistently estimate the causal effect of the exposure on the outcome.

### Clinical samples

Twelve participants were recruited for this study from the First Affiliated Hospital of Shantou University Medical College, comprising 6 RA patients and 6 OA patients. The age and sex information of the participants can be found in **Supplementary Table S1**. Synovial tissue samples were collected from each participant during joint surgery. Our study protocol was approved by the Ethics Committee of the First Affiliated Hospital of Shantou University Medical College (Approval No. B-2024-060). Informed consent was obtained from all enrolled patients.

### RNA isolation and quantitative real-time polymerase chain reaction

Total RNA was extracted from tissue samples using TRIzol (TIANGEN, China) according to the manufacturer’s instructions. RNA was then reverse transcribed into complementary DNA (cDNA) using cDNA Synthesis SuperMix for qPCR (YEASEN, China). qRT-PCR reactions were conducted using Advanced qPCR SYBR Green Master Mix (YEASEN, China). β-Actin was used as an internal control, and relative mRNA quantification was calculated using the 2^−ΔΔCt^ method. Primer sequences used for qRT-PCR are listed in **Supplementary Table S2**.

### Western blot

Total protein was extracted from synovial tissue using RIPA buffer (YEASEN, China) supplemented with protease and phosphatase inhibitors (P002, NCM Biotech, China). Briefly, protein samples were separated by 10% SDS-polyacrylamide gel electrophoresis and transferred onto PVDF membranes. After blocking with 5% non-fat milk, the membranes were incubated overnight at 4°C with primary antibodies: GAPDH (1:10000, 10494-1-AP, Proteintech, USA) and CKAP2 (1:2000, 25486-1-AP, Proteintech, USA), followed by incubation with secondary antibodies at room temperature for 2 hour. Target proteins were detected using an ECL kit (4AW011-200, 4A Biotech, China).

### Hematoxylin and eosin staining and immunohistochemistry

Synovial tissue was fixed in 4% paraformaldehyde, embedded in paraffin, and sectioned into 4 μm-thick slices. The tissue sections were deparaffinized and dehydrated in xylene. For H&E staining, sections were stained with hematoxylin (G1100, Solarbio) and eosin (G1140, Solarbio). IHC was performed using an IHC Kit (KIT-9710, MXB Biotechnologies, China). The primary antibody used was CKAP2 (1:800, PS13605M, Abmart, China). Sections were stained using a DAB Detection Kit (DAB-0031, MXB, China) and counterstained with hematoxylin. Neutral resin was applied to seal the sections, and slides were imaged in the Department of Pathology of Shantou Central Hospital using a digital slide scanner (Pannoramic SCAN, 3DHISTECH Ltd, Hungary).

### Cell culture and transfection

The MH7A (human rheumatoid arthritis synovial cell line) and HFLS-RA cells (human fibroblast-like synoviocytes: rheumatoid arthritis) were purchased from Shanghai Guan&Dao Biological Engineering Co., Ltd. (Shanghai, China). The passage number of all cell lines used for the experiments was no greater than 10. All cell lines were maintained in DMEM (Gibco, USA) supplemented with 10% fetal bovine serum (Gibco, USA), at 37°C under 5% CO_2_. For CKAP2 knockdown, shRNA sequences targeting CKAP2 or a scramble sequence were cloned into an pLKO.1-puro lentiviral cloning vector (Guangzhou IGE Biotechnology Co., Ltd., China). The shRNA and scramble sequences are shown in **Supplementary Table S3**. The virus was packaged in HEK293T cells after transfection with lentiviral packaging vectors using EZ Cell Transfection Reagent II (Shanghai Life-iLab Biotech, China), and then used to infect MH7A and HFLS-RA cells. Transfected cells were selected with puromycin (2 µg/mL) for one week, and expression levels of the target RNA were confirmed by qRT-PCR.

### CCK-8 assay

Cell proliferation was assessed using the Cell Counting Kit-8 (CCK-8) assay (YEASEN, China) according to the manufacturer’s protocol. RA cells were seeded in 96-well plates at a density of 3,000 cells per well. Once the cells had adhered, CCK-8 solution (10 µL) was added to 90 µL of serum-free medium at 0, 24, 48, 72, and 96 hours, followed by a 2-hour incubation at 37°C. Absorbance at 450 nm was measured to determine the optical density (OD) values. Cell proliferation was calculated as follows: cell proliferation = absorbance value at each time point/absorbance value at 0 hours.

### Wound healing assay

Cells were digested with trypsin, counted, and seeded at a density of 1 × 10^7^ cells per well in a 6-well plate, allowing them to grow until confluence. A scratch wound was made using a 200 µL pipette tip. Closure of the wound was monitored at 0, 24, and 48 or 72 hours to assess cell migration.

### Transwell assay

A transwell assay was used to measure cell invasion and migration. Cells were digested with trypsin, and 4 × 10^4^ cells were seeded into the upper chamber of a transwell insert containing serum-free culture medium (for the migration assay) or pre-coated with Matrigel (for the invasion assay). The lower chamber was filled with complete medium to stimulate cell migration. After 48 hours of incubation, the medium and non-migrated or non-invaded cells in the upper chamber were removed. The remaining cells were fixed and stained with 0.1% crystal violet. Migration or invasion was quantified by counting the cells in five random fields at 200X magnification on each membrane.

### Statistical analysis

Data were analyzed using R software (version 4.3.0) and GraphPad Prism (version 9.5.1). Continuous variables were compared using *t*-tests or Wilcoxon tests, while categorical variables were evaluated using chi-square tests. Statistical significance was defined as *p* < 0.05 (two-sided).

## Results

### Identification of DEGs between RA and healthy controls and functional enrichment analysis

**Figure 1:**
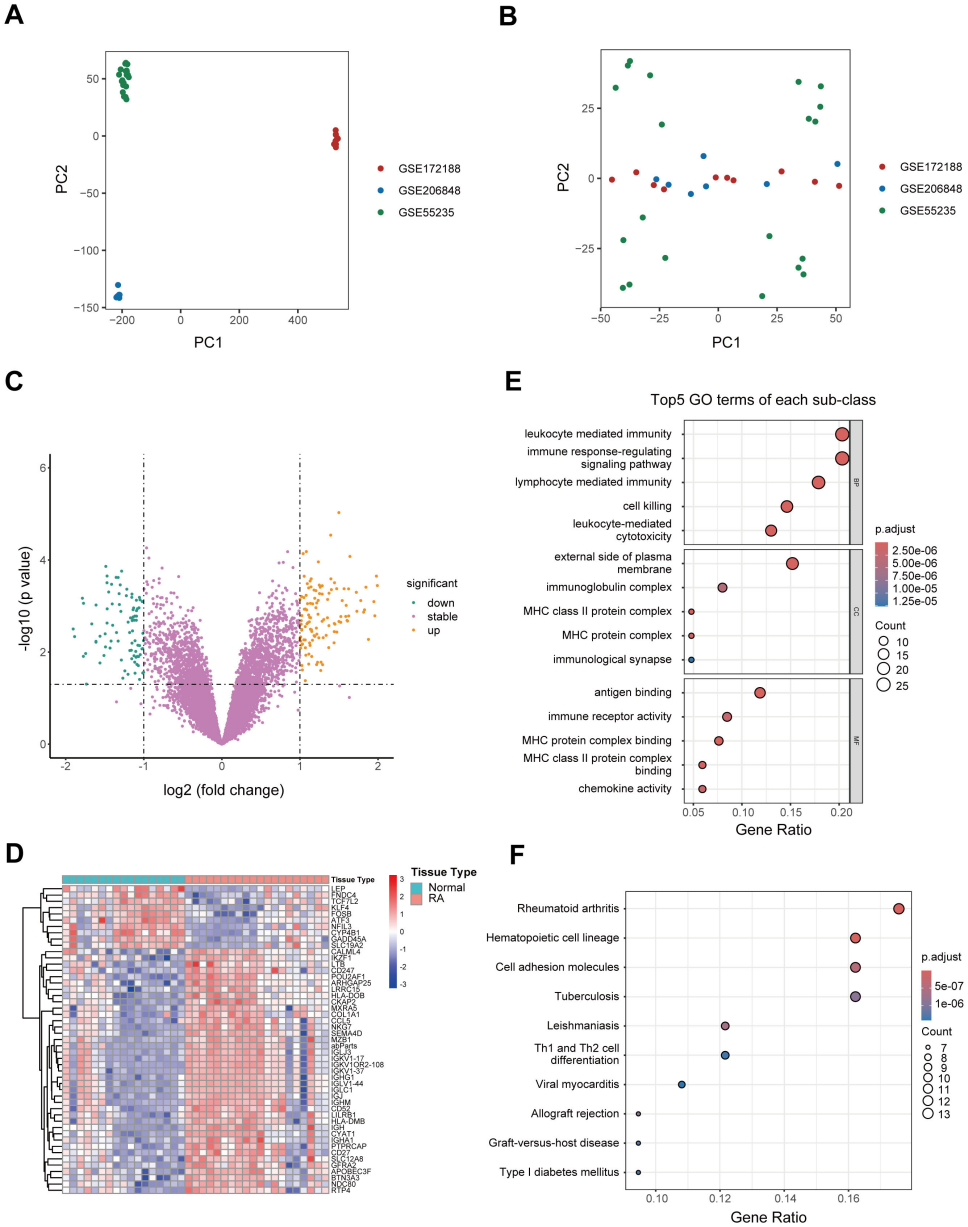
Identification of DEGs and functional enrichment analysis. **(A, B)** PCA plots illustrating the efficiency of batch effect removal [**(A)** before; **(B)** after]. **(C)** Volcano plot depicting the expression of DEGs between RA patients and healthy controls. **(D)** Heatmap illustrating the top genes characterized by the highest standard deviation changes between RA patients and healthy controls. **(E)** Bubble plot showing the top 5 upregulated GO terms in RA patients relative to healthy controls. **(F)** Bubble plot depicting significantly upregulated KEGG pathways in RA patients compared to healthy controls. DEG, Differentially expressed gene; GO, Gene Ontology; KEGG, Kyoto Encyclopedia of Genes and Genomes; PCA, Principal component analysis; RA, Rheumatoid arthritis.

Microarray datasets GSE206848, GSE55235, and GSE172188 were obtained from the GEO database, comprising a total of 20 RA patients and 17 healthy controls. After rigorous quality control, the batch effect was effectively eliminated, ensuring a robust foundation for subsequent analyses (**Figures 1A, B**). Differential expression gene analysis revealed 242 DEGs in RA, with 146 upregulated genes, and 96 downregulated genes. Volcano plots and heatmaps further highlighted the differences in gene expression profiles between RA patients and healthy controls (**Figures 1C, D**). Functional enrichment analysis was conducted on the 146 upregulated genes. GO enrichment analysis revealed that these genes were enriched in pathways related to white blood cell-mediated immunity, immunological synapse formation, immunoglobulin complex, and chemokine activity, indicating their potential involvement in immune responses. In KEGG enrichment analysis, the most significantly enriched pathway was associated with RA, with additional pathways related to Th1 and Th2 cell differentiation and type 1 diabetes, all of which are closely linked to immune responses or immune-related diseases (**Figures 1E, F**).

### Identification of hub genes in RA using WGCNA, RF and LASSO regression analysis

**Figure 2:**
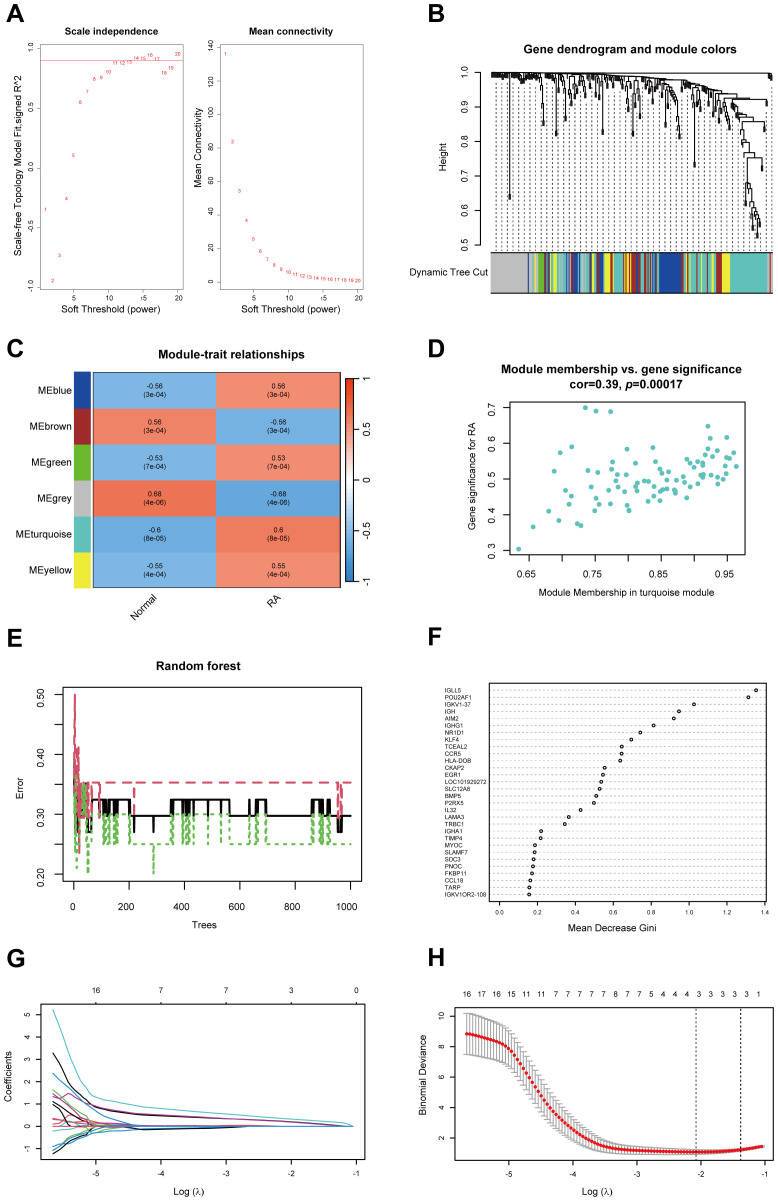
Identification of hub genes in RA using WGCNA, RF and LASSO regression. **(A)** Soft threshold power (left) and mean connectivity (right) of WGCNA. **(B)** Cluster dendrogram derived from WGCNA. **(C)** Heatmap depicting the relationship between modules and RA. **(D)** Scatter plot between gene significance and module members in the turquoise module. **(E)** Confidence intervals for error rates in the RF models within the training group. **(F)** Feature importance plot illustrating the relative importance of genes in the RF model. **(G)** Path diagram of LASSO coefficients for hub genes associated with RA identified through the RF model. **(H)** LASSO regression cross-validation curve with optimal λ values determined by 10-fold cross-validation. WGCNA, Weighted gene co-expression network analysis; RA, Rheumatoid arthritis; RF, Random forest; LASSO, Least absolute shrinkage and selection operator.

To identify hub genes associated with RA, we performed WGCNA on the DEGs between healthy and RA synovial tissues. A soft threshold of 16 was set to ensure a scale-free network distribution (**Figure 2A**). By evaluating gene correlations, we constructed a hierarchical clustering dendrogram, revealing 6 distinct gene modules with similar co-expression patterns (**Figure 2B**). The “turquoise” module exhibited a correlation coefficient of 0.60 and a *p* of 8 × 10–^5^ with the RA, indicating a strong association between the module’s gene expression and RA (**Figure 2C**). Furthermore, within the “turquoise” module, a significant correlation was observed between gene significance and module membership, with a correlation coefficient of 0.39 and a *p* of 0.00017 (**Figure 2D**). As a result, the “turquoise” module was identified as a key module associated with RA.

To further identify hub genes in RA, we applied the RF method and identified 56 core genes (**Figures 2E, F**). Subsequently, LASSO regression analysis was conducted, leading to the identification of 3 hub genes: *CKAP2*, *POU2AF1*, and *HLA-DOB*, all of which were selected as hub genes associated with RA (**Figures 2G, H**).

### Evaluation and validation of hub genes

**Figure 3:**
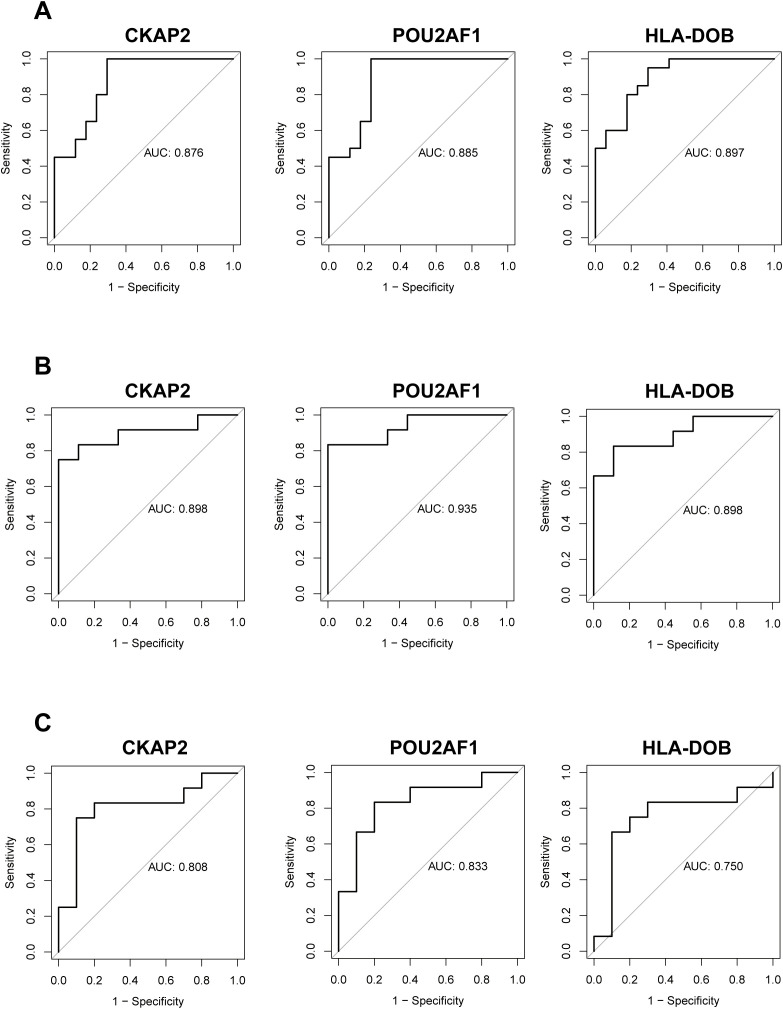
Evaluation and validation of hub genes. **(A)** ROC analysis of the three hub genes in RA patients compared to healthy controls within the training group. **(B)** ROC analysis of the same hub genes in RA patients vs. healthy controls in the validation group. **(C)** ROC analysis of the hub genes in RA patients vs. OA patients in the validation group. OA, Osteoarthritis; RA, Rheumatoid arthritis; ROC, Receiver operating characteristic.

To assess the diagnostic efficacy of the identified hub genes, we performed ROC curve analysis. In the training set, the AUC values were 0.876 for *CKAP2*, 0.885 for *POU2AF1*, and 0.897 for *HLA-DOB* (**Figure 3A**). In the validation set, compared to healthy controls, the AUC values were 0.898 for *CKAP2*, 0.935 for *POU2AF1*, and 0.898 for *HLA-DOB* (**Figure 3B**). Additionally, to evaluate the specificity of the hub genes for RA, we compared RA to OA in the validation set and calculated the AUC values for the hub genes. The results showed AUC values of 0.808 for *CKAP2*, 0.833 for *POU2AF1*, and 0.750 for *HLA-DOB* (**Figure 3C**). These findings indicate that the 3 hub genes possess high diagnostic value for RA patients, regardless of whether they are compared with healthy controls or OA controls. Furthermore, we compared the expression profiles of the hub genes in RA patients, healthy controls, and OA controls in both the training and validation sets. The results demonstrated that these hub genes were expressed at higher levels in RA patients (**Supplementary Figures S1A–C**).

### Immune infiltration and GSVA analysis of hub genes

**Figure 4:**
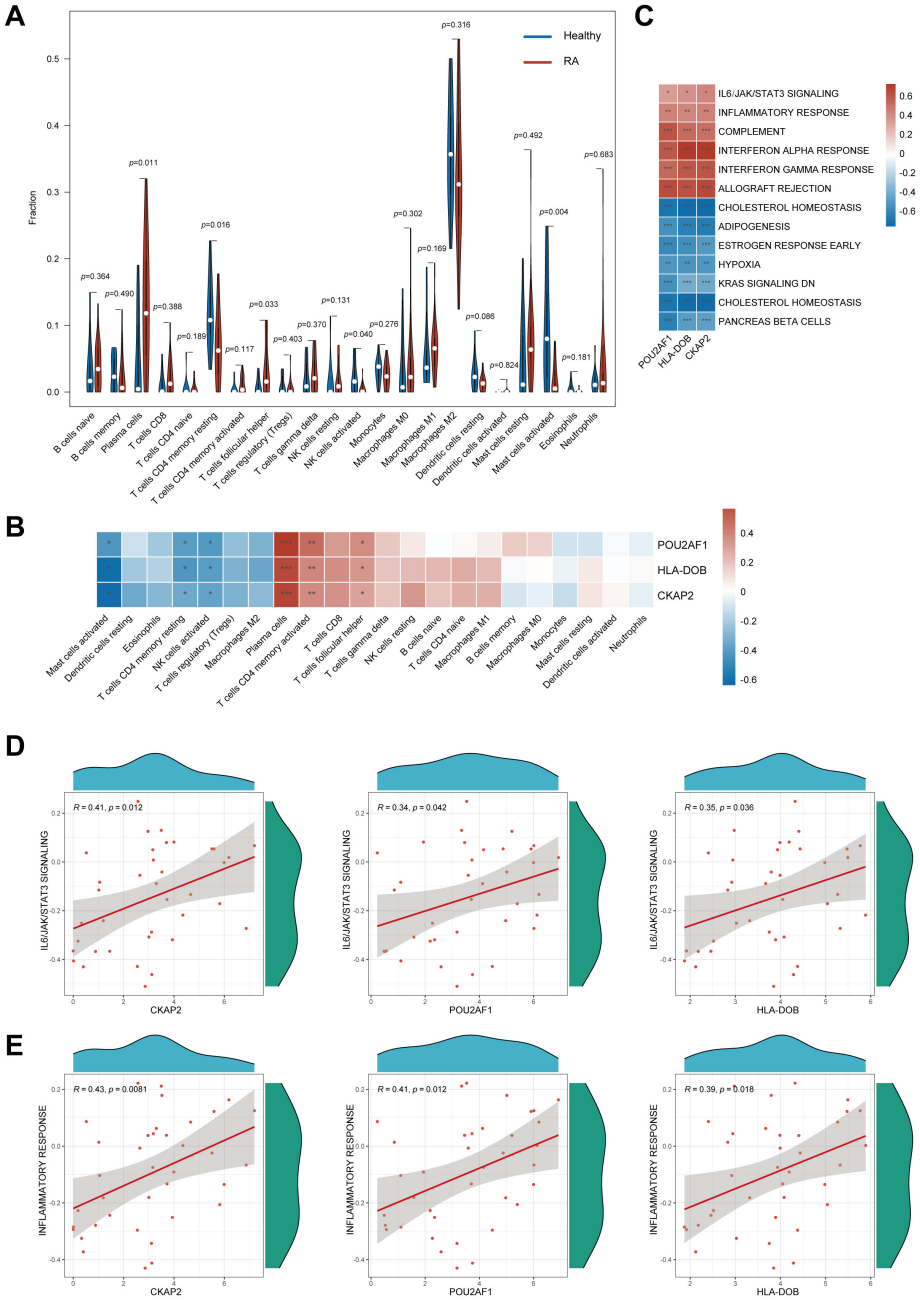
Immune infiltration analysis and GSVA of hub genes. **(A)** Immune cell infiltration profiles between RA patients and normal controls. **(B)** Association between the three hub genes and various immune cell infiltrates. **(C)** GSVA of hub genes. **(D)** Association of the 3 hub genes with IL6/JAK/STAT3 signaling pathways. **(E)** Association of the 3 hub genes with inflammatory response. GSVA, Gene set variation analysis; RA, Rheumatoid arthritis; *, *p* < 0.05; **, *p* < 0.01; ***, *p* < 0.001.

The CIBERSORT algorithm revealed significant differences in immune cell infiltration between RA patients and healthy controls in the synovium. Specifically, RA patients exhibited markedly increased infiltration of plasma cells (*p* = 0.011) and follicular helper T (Tfh) cells (*p* = 0.033), while healthy controls showed higher levels of resting memory CD4+ T cells (*p* = 0.016), activated NK cells (*p* = 0.040), and activated mast cells (*p* = 0.004). A relatively large proportion of M2 macrophages were found in both RA patients and healthy controls, but they were not statistically different (**Figure 4A**). Further analysis of immune cell infiltration in RA revealed associations between 3 hub genes and specific immune cell types. These genes were positively correlated with the infiltration of plasma cells, Tfh cells, and activated CD4+ T cells, while showing negative correlations with the infiltration of resting memory CD4+ T cells, activated NK cells, and activated mast cells (**Figure 4B**).

GSVA demonstrated that *CKAP2*, *POU2AF1*, and *HLA-DOB* were associated with pathways, such as IL6/JAK/STAT3 signaling, and involved in inflammatory response, complement activation, and interferon response (**Figure 4C**). Scatter plots with Spearman correlation analysis were performed to compare the relationship between the hub genes and the IL6/JAK/STAT3 signaling pathway, as well as the inflammatory response (**Figures 4D, E**).

### MR analysis to identify the causal relationship between *CKAP2* and RA

**Figure 5:**
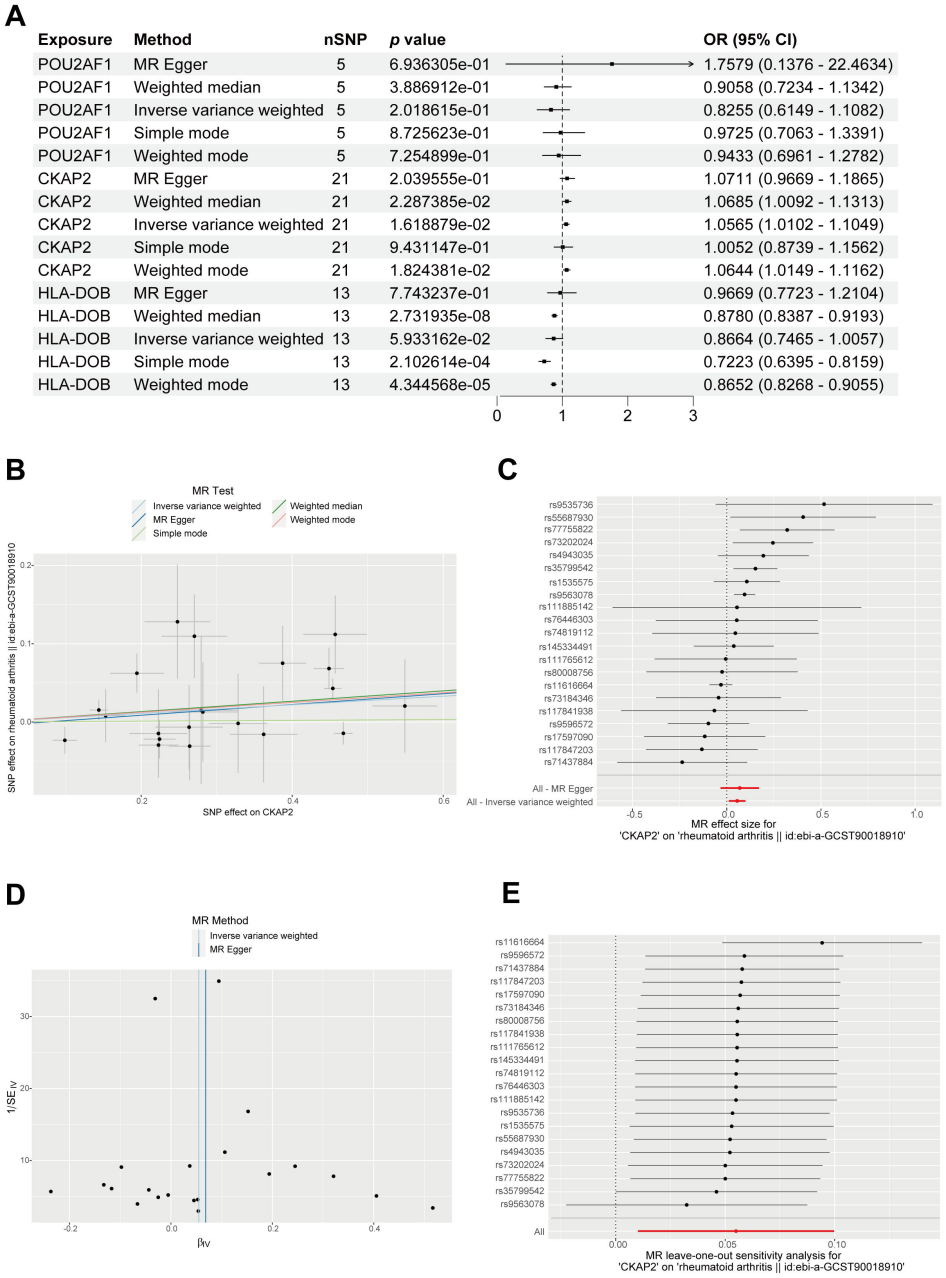
MR analysis identifying the genetically linked gene *CKAP2*. **(A)** Forest plot displaying causal effects between the 3 hub genes and RA through multiple MR methods. **(B)** Scatter plot, employing 5 methods, illustrating causal estimates of *CKAP2* on RA. **(C)** Forest plot for MR pleiotropy testing. **(D)** MR funnel plot showcasing IVW and MR-Egger methods. **(E)** Leave-one-out sensitivity analysis forest plot for MR. IVW, Inverse variance-weighted; MR, Mendelian randomization; RA, Rheumatoid arthritis.

MR analysis integrates GWAS and expression quantitative trait locus (eQTL) data, and was used to investigate the association between RA and the eQTLs of *CKAP2*, *POU2AF1*, and *HLA-DOB*. Results from the IVW, WM, and weighted models consistently supported a positive correlation between *CKAP2* and RA (**Figure 5A**). A scatter plot illustrating the SNP effect size for *CKAP2* and RA shows the SNP effect on *CKAP2* along the x-axis and the SNP effect on RA along the y-axis (**Figure 5B**). MR-Egger analysis revealed no evidence of horizontal pleiotropy (*p* = 0.77, **Figure 5C**, **Supplementary Table S4**). The funnel plot demonstrated no significant heterogeneity among the SNPs (*p* < 0.05, **Figure 5D**, **Supplementary Table S5**). Leave-one-out analysis indicated that individual SNPs did not influence the MR analysis results (**Figure 5E**). These findings support a positive causal relationship between *CKAP2* and RA.

### CKAP2 expression is increased in synovial tissues from RA patients

**Figure 6:**
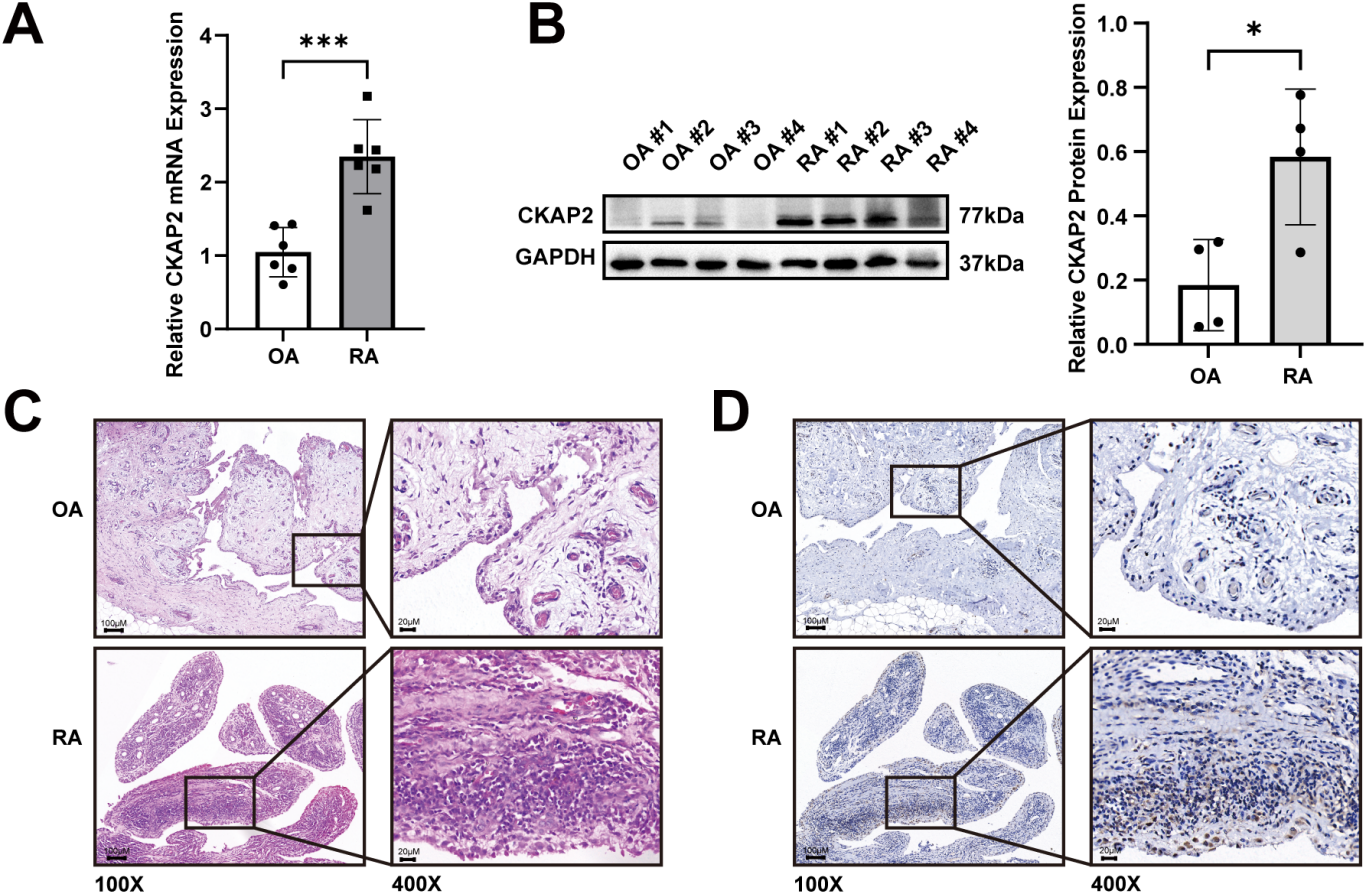
CKAP2 expression is increased in synovial tissues from RA patients. **(A)** *CKAP2* mRNA expression in synovial tissues from RA and OA patients. **(B)** Western blot analysis revealing CKAP2 protein levels in synovial tissues from RA and OA patients. **(C)** H&E staining for CKAP2 in synovial tissues from RA and OA patients. **(D)** IHC staining for CKAP2 in synovial tissues from RA and OA patients. H&E, Hematoxylin and eosin; IHC, Immunohistochemistry; OA, Osteoarthritis; RA, Rheumatoid arthritis. *, *p* < 0.05; ***, *p* < 0.001.

We examined CKAP2 expression levels in synovial tissues from patients with RA and OA. qRT-PCR and western blot analyses revealed an upregulation of CKAP2 mRNA (**Figure 6A**) and protein (**Figure 6B**) in RA synovial tissues. Histopathological analysis using H&E staining showed marked synovial hyperplasia and increased immune cell infiltration in RA compared to OA (**Figure 6C**). IHC confirmed CKAP2 expression in both RA and OA synovium, with higher expression in RA synovium, compared to OA synovium (**Figure 6D**). To determine whether *CKAP2* expression is specific to synovial tissue, we analyzed *CKAP2* expression levels in peripheral blood mononuclear cells from 232 RA patients and 43 healthy controls using the GSE93777 dataset. No significant difference was observed between the two groups (**Supplementary Figure S2**).

### CKAP2 promotes the proliferation, migration and invasion of MH7A and HFLS-RA cells

We knocked down CKAP2 in MH7A and HFLS-RA cells, using shRNA, and validated the extent of silencing by qRT-PCR. The results indicated that CKAP2 expression was reduced in the shCKAP2 groups compared to the scramble group.

**Figure 7:**
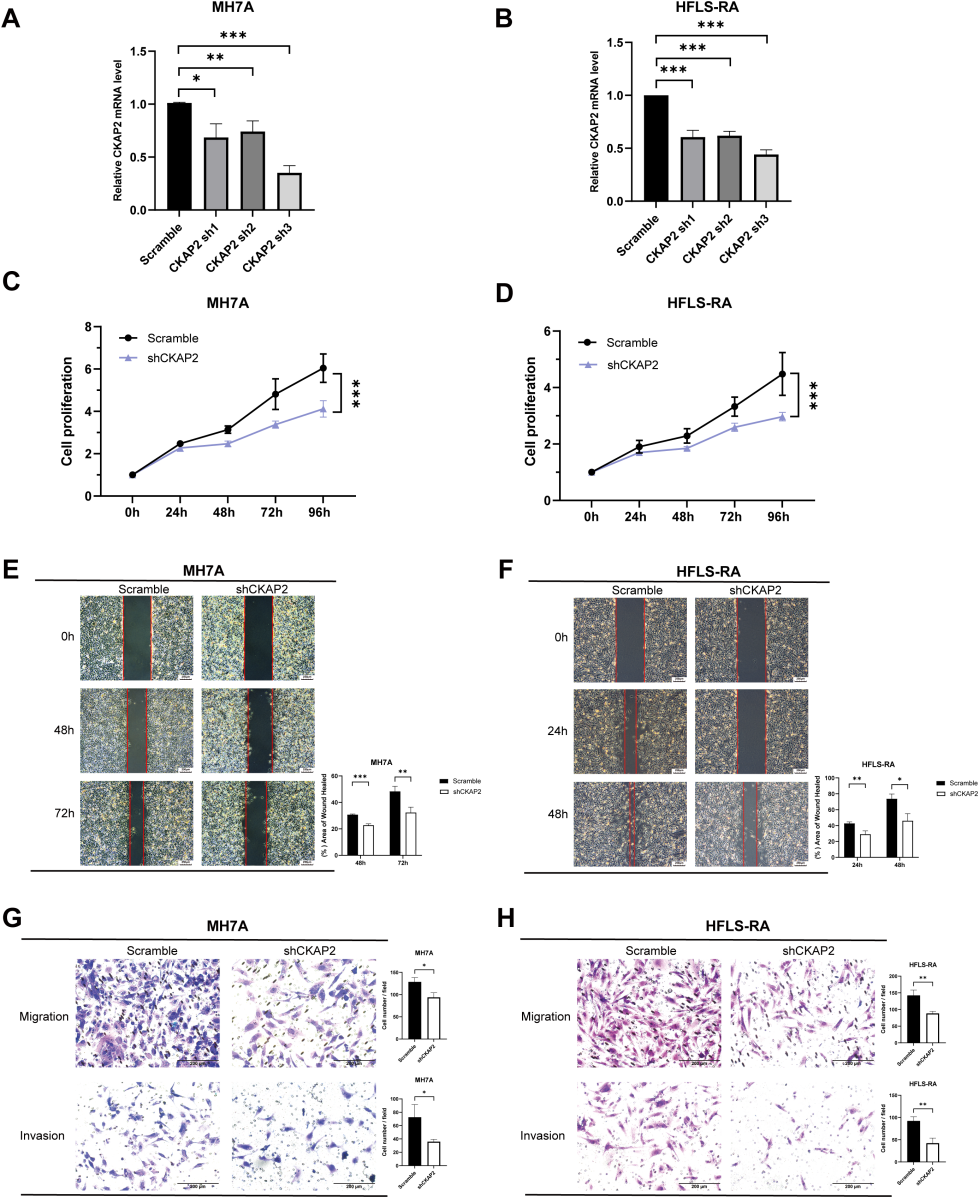
Knockdown of CKAP2 inhibits MH7A and HFLS-RA cell proliferation, migration, and invasion. **(A, B)** qRT-PCR showing *CKAP2* mRNA levels in MH7A and HFLS-RA cells after infection with either scrambled or shCKAP2 lentivirus. **(C, D)** CCK-8 assay demonstrating inhibition of cell proliferation following CKAP2 knockdown in MH7A and HFLS-RA cells. **(E, F)** Wound-healing assay indicating reduced migration after CKAP2 knockdown in MH7A and HFLS-RA cells. **(G, H)** Transwell assay revealing inhibited migration and invasion after CKAP2 knockdown in MH7A and HFLS-RA cells. *, *p* < 0.05; **, *p* < 0.01; ***, *p* < 0.001.

Meanwhile, CKAP2 sh3 showed the superior silencing efficiency and was thus selected for subsequent experiments. (**Figures 7A, B**).

To assess the effect of CKAP2 on cell proliferation, we performed a CCK-8 assay. shCKAP2 reduced the proliferation of MH7A and HFLS-RA cells compared to the scramble group (**Figures 7C, D**, both *p* < 0.001), suggesting that CKAP2 knockdown suppressed cell proliferative ability.

To evaluate the impact of CKAP2 on migration and invasion, we conducted wound-healing and transwell migration and invasion assays. Wound-healing assays showed wound surface areas for the shCKAP2 group were larger than for the scramble group in both MH7A and HFLS-RA cells (**Figures 7E, F**). Similarly, transwell assays demonstrated that knockdown of CKAP2 inhibited both migration and invasion of MH7A and HFLS-RA cells (**Figures 7G, H**). Therefore, these results suggest that CKAP2 promotes the migration and invasion of MH7A and HFLS-RA cells.

## Discussion

Based on bioinformatics analysis combined with machine learning algorithms and MR analysis, we identified and validated *CKAP2* as a signature gene of RA. Additionally, our study demonstrates that CKAP2 is involved in RA pathogenesis by promoting the proliferation, migration, and invasion of FLS, as demonstrated by clinical tissue samples and cellular functional assays.

In this study, we retrieved and merged 3 microarray cohorts. Using bioinformatics analysis and machine learning algorithms, we identified 3 hub genes associated with RA: *CKAP2*, *POU2AF1*, and *HLA-DOB*. These genes demonstrated strong diagnostic efficacy in both the training set and external validation set, with significantly higher expression levels in RA patients. This indicates that our approach to identifying hub genes is both reliable and feasible. Previous studies have suggested that these hub genes may be associated with the pathogenesis of RA. Research by Levels et al. has shown that the POU2AF1 were significantly elevated in the synovial tissue of RA patients and revealed that it may be a key transcription factor in the activation of pathogenic B cells (30, 31). Teitell et al. indicated that POU2AF1, as a B lymphocyte-specific coactivator, regulates the expression of immunoglobulins and other immune-related genes, and is involved in immune and inflammatory responses (32). Afroz et al. conducted a meta-analysis and found that HLA-DOB is significantly upregulated in peripheral blood mononuclear cells in RA patients. HLA-DOB, a B cell lineage, MHC-II-related molecule has been reported to exhibit strong immunogenicity in human T cells (33). Kang et al. found that the HLA-DOB_232–240_ epitope could serve as an immunotherapeutic target for multiple myeloma (34). Therefore, HLA-DOB might also be a potential therapeutic target for controlling inflammation in RA. Currently, there are no reports on the role of CKAP2 in RA, but substantial evidence from previous studies indicated that CKAP2 was associated with cell proliferation, migration, and invasion, which are key factors in joint destruction caused by FLS in RA (24, 25, 35–37).

Immune infiltration analysis suggests that plasma cells and Tfh cells are the primary infiltrating cell types in the RA synovium, potentially playing a pivotal role in the pathogenesis of RA. These findings are consistent with those of previous studies. Zhang et al. utilized single-cell RNA sequencing analysis to demonstrate the expansion of autoimmunity-associated B cells (ABCs) in the synovium of RA patients (38). Similarly, Qin et al. confirmed the expansion of ABCs in both the peripheral blood and synovial tissue of RA patients, suggesting a potential role of ABCs in inflammatory arthritis (39). Furthermore, Li et al. reported an increased number of ABCs, in both mouse models of inflammatory arthritis and human peripheral blood, which are recruited to inflamed joints through chemotactic mechanisms, promoting the progression of chronic synovitis by secreting self-reactive antibodies (40). Additionally, Tfh cells, a subpopulation of CD4+ T cells, promote the activation of autoreactive B cells and the production of high-affinity antibodies. Increased numbers of Tfh cells are strongly associated with exacerbation of rheumatoid arthritis (41–43). Furthermore, we observed a relatively large proportion of M2 macrophages in both RA and healthy controls, which is still an interesting phenomenon even though there was no statistical difference between the two groups. It has been found that an increased proportion of M2 macrophages in RA synovial tissues is associated with lower RA disease activity and synovial inflammation, with anti-inflammatory and homeostatic functions (44), whereas synovial macrophages in healthy synovial tissues are predominantly CD206-positive macrophages, the M2 macrophages, which play an important role in the maintenance of tissue homeostasis (45). A systematic review found no significant difference in the proportion of M2 macrophages in synovial tissue and PBMC between RA and healthy controls, a finding consistent with our results (46).

To further investigate the role of hub genes in RA, we employed MR analysis to examine the causal relationship between hub genes and RA. The results indicated that upregulation of *CKAP2* is associated with RA. CKAP2 expression is higher in RA synovial tissue compared to OA synovial tissue. H&E staining showed increased immune cell infiltration in RA compared to OA, as RA is an autoimmune disease characterized by synovial inflammation and hyperplasia as the core pathological changes, while OA is primarily considered a degenerative disease mainly associated with the aging process (47). The number of inflammatory cells in OA synovial tissue is fewer than to RA synovial tissue, which is consistent with the findings of Lange-Brokaar et al. (48). Additionally, cellular functional assays suggested that CKAP2 plays a central role in RA pathogenesis by promoting the proliferation, migration, and invasion of fibroblast-like synovial cells. Numerous studies have suggested that CKAP2 promotes tumor cell proliferation, migration, and invasion through the JAK/STAT3 signaling pathway or the FAK-ERK2 pathway, which could contribute to poor disease prognosis (17, 35, 49, 50), as observed in cancers such as gastric adenocarcinoma, hepatocellular carcinoma, cervical carcinoma, and breast carcinoma (20, 22–24). Moreover, GSVA indicated that CKAP2 may influence RA through the IL-6/JAK/STAT3 pathway, which is consistent with previous research on CKAP2. Zhang et al. demonstrated that the levels of phosphorylated JAK2 and STAT3 are lower in CKAP2 knockdown cells compared to control cells, inducing G0/G1 arrest and apoptosis in osteosarcoma cells (35). Jin et al. showed that inhibiting CKAP2-mediated FAK and STAT3 phosphorylation signaling could suppress the proliferation, adhesion, and migration of triple-negative breast cancer cells (17). Zhang et al. found that hypermethylation-regulated silencing of miR-9, along with CKAP2, activates the IL-6/JAK/STAT3 pathway, potentially contributing to cancer cell growth, migration, and malignant transformation (51). Therefore, CKAP2 may promote the onset and progression of RA by activating the IL-6/JAK/STAT3 pathway.

To summarize, our study may contribute to the molecular diagnosis and targeted therapy of RA. However, the datasets used in our study are mainly from Europe, which may limit the generalizability of our findings. Future multicenter and multiregional data are necessary to confirm our conclusions. Additionally, the relatively small sample size may reduce the statistical power and generalizability of the findings.

## Conclusion

This study employed bioinformatics analysis, combining machine learning algorithms with MR analysis, to identify and validate *CKAP2* as a signature gene of RA. Clinical tissue samples and cellular function experiments confirmed that CKAP2 is highly expressed in the RA synovium and promotes the proliferation, migration, and invasion of FLS, contributing to RA pathogenesis. As a novel biomarker, CKAP2 may provide valuable insights into molecular diagnosis and targeted therapies for RA.

## Data Availability

The datasets presented in this study can be found in online repositories. The names of the repository/repositories and accession number(s) can be found in the article/**Supplementary Material**.
