# Supplementary material for: Identification and validation of CKAP2 as a novel biomarker in the development and progression of rheumatoid arthritis
Source: Front Immunol. 2025 Jun 25;16:1606201. doi: 10.3389/fimmu.2025.1606201 (PMC12238030; doi:10.3389/fimmu.2025.1606201)
Supplement: Supplementary file 1 [file Table1.docx]

**Supplementary Material**


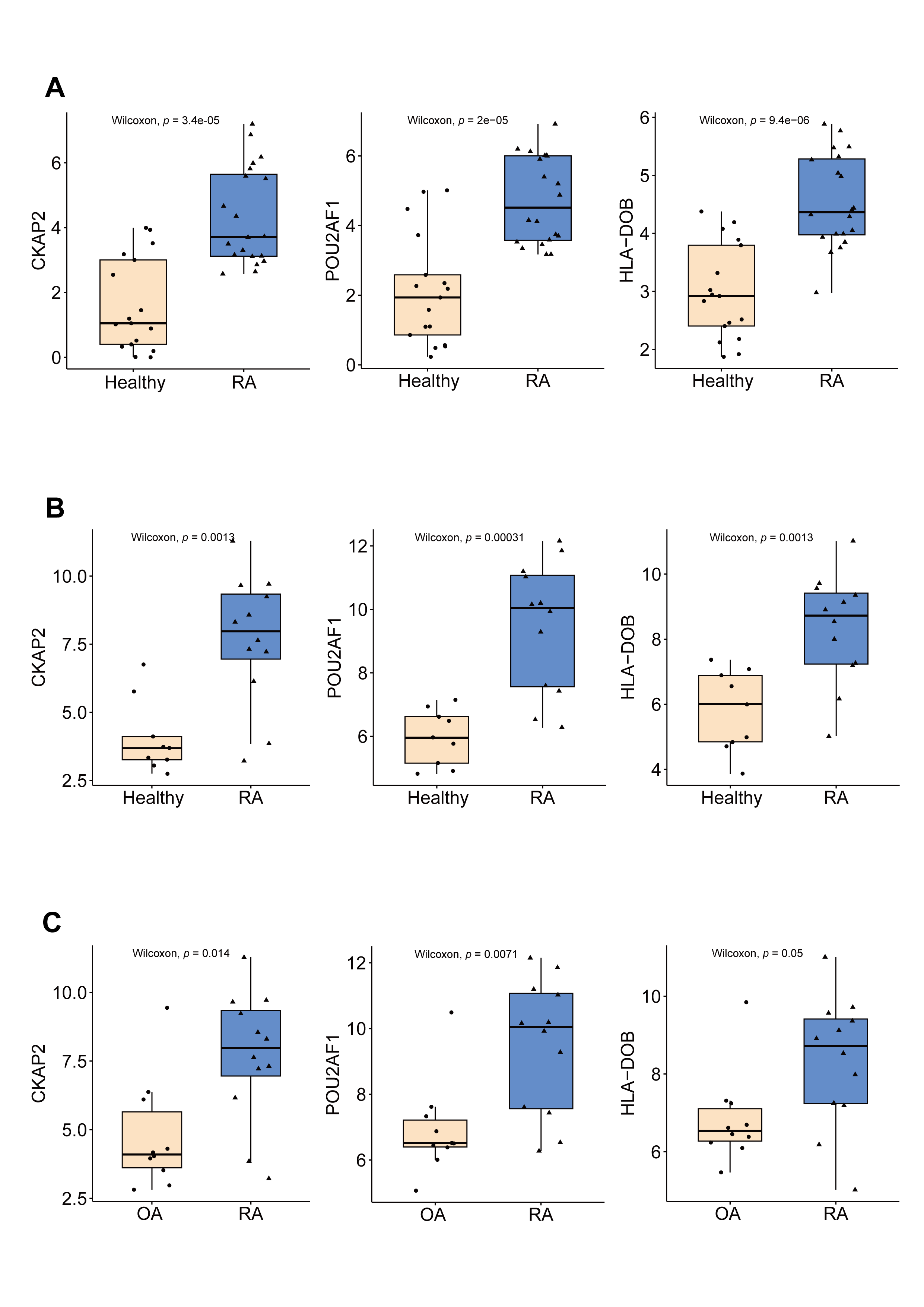


**Figure S1. Expression levels of hub genes.** (A) Expression levels of the three hub genes in RA patients versus healthy controls within the training group. (B) Expression levels of the three hub genes in RA patients versus healthy controls in the validation group. (C) Expression levels of the three hub genes in RA patients compared to OA in the validation group. OA, Osteoarthritis; RA, Rheumatoid arthritis.


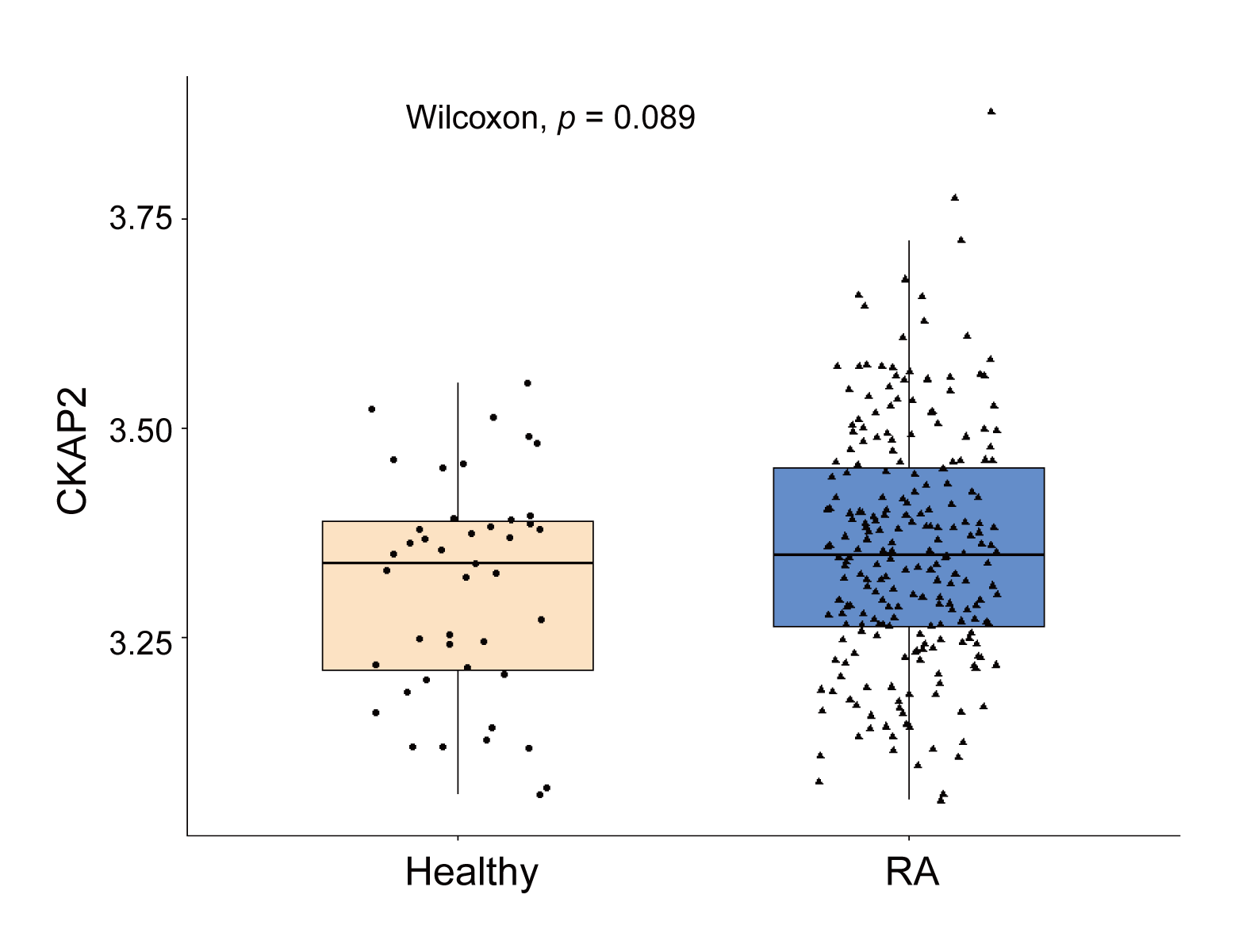


**Figure S2. *CKAP2* expression levels in peripheral blood mononuclear cells.** No significant difference in *CKAP2* expression was observed between Healthy controls and RA patients.

**TableS1. Age and gender distribution of patients**

|  | **Group** | **Gender (male/female)** | **Age (years)** |
| --- | --- | --- | --- |
| **Training group** |  |  |  |
| GSE206848 | Healthy(n=7) | 5/2 | 50-74 (range) |
| GSE55235 | Healthy(n=10) | Not available | Not available |
|  | RA(n=10) | Not available | Not available |
| GSE172188 | RA(n=10) | 4/6 | 56.25±16.68 |
| **Validation group** |  |  |  |
| GSE12021 | RA(n=12) | 3/9 | 64.83±10.43 |
|  | OA(n=10) | 2/8 | 71.9±6.44 |
|  | Healthy(n=9) | 7/2 | 50.22±21.91 |
| **Our clinical samples** | RA(n=6) | 0/6 | 62.17±7.03 |
|  | OA(n=6) | 0/6 | 60.5±15.19 |

**Table S2. List of primer sequences.**

| **Gene** | **Forward (5'-3')** | **Reverse (5'-3')** |
| --- | --- | --- |
| CKAP2 | CCGTTGACCAGCGAAGACATA | CACTCACTCAGACGAGCTTTTC |
| β-Actin | TTGGCAATGAGCGGTTCC | AGACAGCACTGTGTTGGC |

**Table S3. List of shRNA sequences.**

| **shRNA** | **Sequence** |
| --- | --- |
| CKAP2 sh1 | 5′-CGACCTCCTATTAGAAGTCAT-3′ |
| CKAP2 sh2 | 5′-CCCTGTTCACTTTACTAAATA-3′ |
| CKAP2 sh3 | 5′-TATGAGGCTGATACAACATAA-3′ |
| Scramble shRNA | 5′-CCTAAGGTTAAGTCGCCCTCG-3′ |

**Table S4. Pleiotropy analysis for RA**

| **Outcome** | **Exposure** | **Egger intercept** | **SE** | ***P-value*** |
| --- | --- | --- | --- | --- |
| Rheumatoid arthritis | POU2AF1 | -0.065908194 | 0.112432241980948 | 0.598925871332379 |
| Rheumatoid arthritis | CKAP2 | -0.005121342 | 0.0174090329237187 | 0.771812156098046 |
| Rheumatoid arthritis | HLA-DOB | -0.038962925 | 0.0310171361419057 | 0.235076679824593 |

**Table S5. Heterogeneity of exposure and outcome in RA**

| **Outcome** | **Exposure** | **Method** | **Q** | **Q df** | **Q *P-value*** |
| --- | --- | --- | --- | --- | --- |
| Rheumatoid arthritis | POU2AF1 | MR Egger | 12.5929314147426 | 3 | 0.00560495718028118 |
| Rheumatoid arthritis | POU2AF1 | Inverse variance  -weighted | 14.0353877131288 | 4 | 0.00718296502459663 |
| Rheumatoid arthritis | CKAP2 | MR Egger | 34.9341722059213 | 19 | 0.0142239399346911 |
| Rheumatoid arthritis | CKAP2 | Inverse variance  -weighted | 35.0932886687748 | 20 | 0.0196130173183284 |
| Rheumatoid arthritis | HLA-DOB | MR Egger | 171.712882065932 | 11 | 5.2388475732982e-31 |
| Rheumatoid arthritis | HLA-DOB | Inverse variance  -weighted | 196.345462783201 | 12 | 1.85078110052429e-35 |
